# "T-bet"-ing on autoimmunity variants

**DOI:** 10.1371/journal.pgen.1006924

**Published:** 2017-09-07

**Authors:** Michelle L. T. Nguyen, Dimitre R. Simeonov, Alexander Marson

**Affiliations:** 1 Department of Microbiology and Immunology, University of California, San Francisco, California, United States of America; 2 Diabetes Center, University of California, San Francisco, California, United States of America; 3 Innovative Genomics Institute, University of California, Berkeley, California, United States of America; 4 Biomedical Sciences Graduate Program, University of California, San Francisco, California, United States of America; 5 Department of Medicine, University of California, San Francisco, California, United States of America; 6 UCSF Helen Diller Family Comprehensive Cancer Center, University of California, San Francisco, California, United States of America; 7 Chan Zuckerberg Biohub, San Francisco, California, United States of America; University of Melbourne, AUSTRALIA

During the past decade, genome-wide association studies (GWAS) have linked autoimmune disorders to hundreds of candidate risk variants across the human genome. Yet, our understanding of how these variants confer disease risk has severely lagged behind our ability to identify them. Almost 90% of GWAS hits map to noncoding regions of the genome, which creates a challenge to extract mechanistic insight from the human genetics. Although mounting evidence suggests that the majority of disease-associated SNPs lie in enhancers and may alter transcription factor (TF) occupancy and target gene regulation [1], only a handful of noncoding disease variants have been experimentally validated [2–4]. In a recent issue of *PLOS Genetics*, Soderquest et al. examined how genetic variants might alter the binding landscape of T-bet (encoded by *TBX21*), a critical transcriptional regulator in immune cells [5].

CD4^+^ T cells, which are central in autoimmune pathology, can take on distinct effector functions in the context of inflammation, including Th1, Th2, Th17, and regulatory T cell (Treg) phenotypes. Each subset is defined by characteristic cytokines and cell surface receptors—programs that are driven by master TFs [6]. Disruption of these programs could lead to disease. In patients with Crohn’s disease, T cells from the lamina propria express high levels of T-bet, the master regulator of Th1 cells [7], potentially implicating this TF in inflammatory bowel disease (IBD) pathology.

Soderquest et al. asked whether common noncoding disease variants might contribute to autoimmune disease risk by altering T-bet occupancy and regulation of genomic targets [5]. The authors previously used chromatin immunoprecipitation sequencing (ChIP-seq) to map T-bet genomic binding sites in primary human Th1 cells [8–10]. Here, they found that GWAS risk variants linked with Crohn’s disease, and to a lesser extent ulcerative colitis and celiac disease, preferentially mapped to T-bet-bound Th1 regulatory elements. Interestingly, GWAS variants associated with rheumatoid arthritis and psoriasis were not enriched at T-bet binding sites, raising the possibility that genetic effects on the T-bet program may be disease-specific.

**Fig 1:**
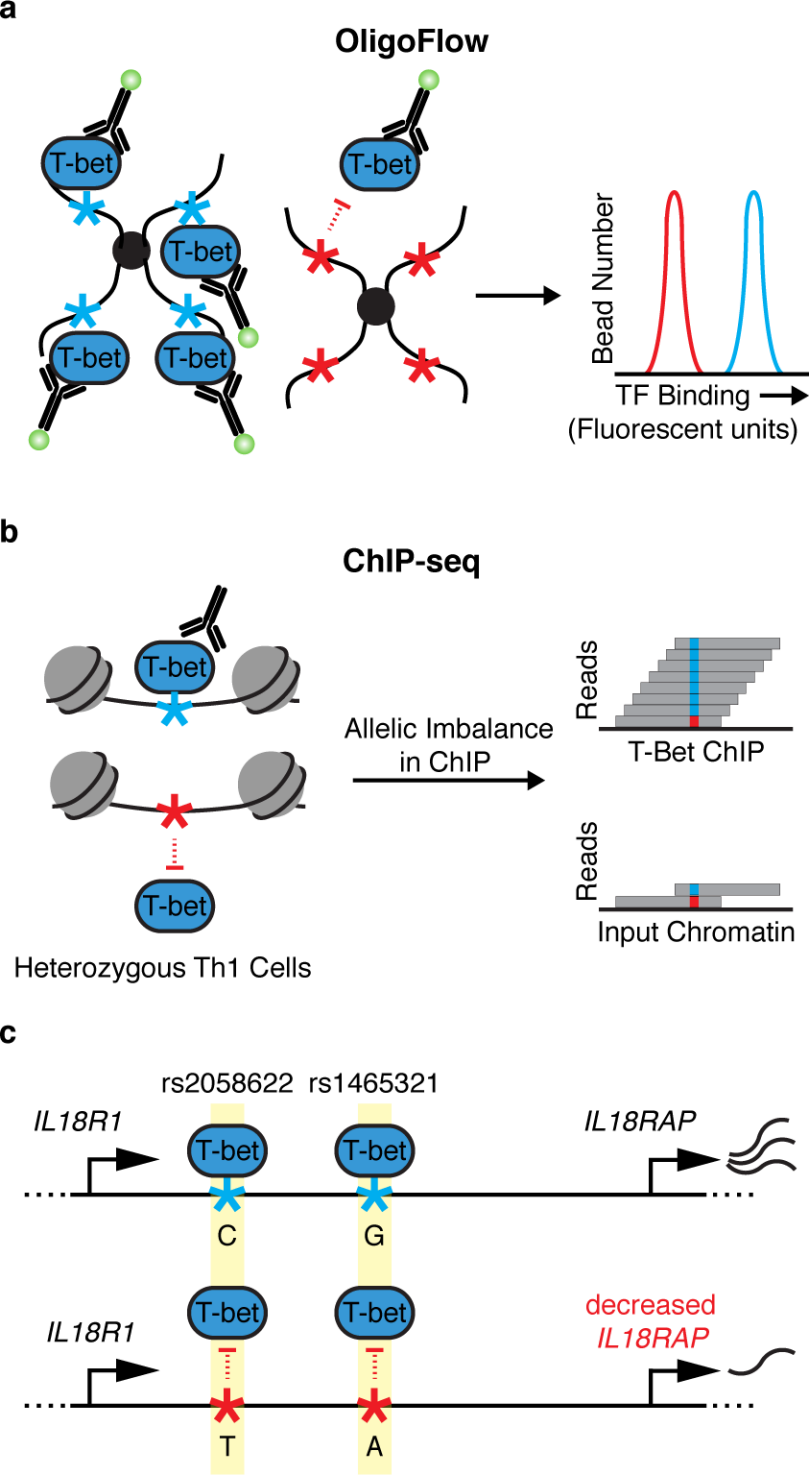
Identifying noncoding variants that impair transcription factor (TF) binding. (a) OligoFlow is a flow-cytometry–based method for rapid screening of DNA variants that affect TF binding. In brief, cell lysate is mixed with bead-coupled oligonucleotides containing a T-bet binding site (light blue asterisk, left panel) or without (red asterisk, right panel) a candidate disease variant. Samples are incubated with a fluorochrome-labeled antibody against T-bet, and binding affinity is measured by flow cytometry. (b) chromatin immunoprecipitation sequencing (ChIP-seq) allows for the assessment of TF binding in its native chromatin context. Soderquest et al., confirmed allelic imbalance of T-bet binding to 2 linked SNPs (rs1465321 and rs2058622) at the immune risk locus *IL18RAP* (c). These SNPs are associated with decreased expression of the gene [11], suggesting that they affect T-bet–dependent regulation of *IL18RAP*. *IL18R1/IL18RAP* locus not drawn to scale.

This enrichment of IBD and celiac risk variants at T-bet binding sites motivated further efforts to dissect the effects of noncoding variants. The authors developed OligoFlow to rapidly screen for variants that alter T-bet binding affinity. This new method relies on mixing cell lysate containing T-bet with bead-conjugated oligonucleotides corresponding to T-bet binding sites with or without a candidate disease-variant. A fluorochrome-labeled antibody against the TF of interest—in this case, T-bet—is then added to quantify the in vitro TF-oligo affinity by flow cytometry (Fig 1A). The authors used OligoFlow to test T-bet binding at a number of variants and demonstrated significant reduction of T-bet binding for 3 of them: rs1465321, rs1006353, and rs11135484. Interestingly, despite exhibiting altered T-bet binding, only 1 of these variants falls in a recognizable T-bet binding motif. The mechanisms by which the other variants alter T-bet binding remain unclear—they could affect noncanonical sequences that directly affect T-bet affinity, or they could influence the binding of neighboring TFs that contribute to T-bet recruitment. OligoFlow is likely to prove useful as a general tool to screen for the effects of sequence variation on DNA–TF interactions.

The authors went on to validate the effects of rs1465321 on T-bet occupancy and target gene regulation in its endogenous chromatin context. They performed ChIP-seq on in vitro polarized primary human Th1 cells from individuals who are heterozygous for rs1465321 and confirmed allelic imbalance of T-bet binding to the SNP as well as a nearby linked SNP (rs2058622) (Fig 1B and 1C). rs1465321 falls within a risk locus for celiac disease, which contains multiple SNPs that are associated with decreased expression of *IL18RAP* [11]. The authors also confirmed that *Il18rap* expression was significantly reduced in T-bet–deficient murine Th1 cells compared with wild-type cells. Taken together, the results highlight a T-bet–dependent gene regulatory circuit disrupted by a common human variant.

The field of human immunogenetics is coming to maturity. High-density genotyping arrays and new statistical algorithms have made it possible to confidently identify causal risk variants. The focus now more than ever is on how to determine the biological significance of disease variants. Soderquest et al. suggest that a subset of risk variants associated with IBD and celiac disease alter binding of the Th1 master regulatory TF T-bet. However, there are still important questions that remain unanswered. Further studies will be needed to examine if the effects of variants are specific to T-bet genome occupancy. Alternatively, there may be sets of key TFs with altered binding profiles contributing to the risk for distinct diseases. In addition, T-bet also contributes to gene regulatory programs in multiple immune cell types, including CD8^+^ T cells, innate lymphoid cells, natural killer (NK) cells, and even subsets of Tregs and Th17 cells [12]. Disease variants may have pleotropic effects on gene regulation in multiple cell types. Along with accelerating the acquisition of human genetic data, mechanistic studies are now possible with advanced chromatin mapping approaches and genome engineering technologies. OligoFlow offers a complementary technology to rapidly test for the effects of noncoding genetic variation. Studies integrating these emerging techniques have the potential to reveal underlying pathological mechanisms and, ultimately, could lead to targeted treatments for common human diseases.
